# Differences in depressive symptoms by rurality in Japan: a cross-sectional multilevel study using different aggregation units of municipalities and neighborhoods (JAGES)

**DOI:** 10.1186/s12942-021-00296-8

**Published:** 2021-09-26

**Authors:** Mariko Kanamori, Masamichi Hanazato, Daisuke Takagi, Katsunori Kondo, Toshiyuki Ojima, Airi Amemiya, Naoki Kondo

**Affiliations:** 1 Department of Health and Social Behavior, Graduate School of Medicine, The University of Tokyo, Bldg. 3, 7-3-1 Hongo, Bunkyo-ku, Tokyo, Japan; 2 Department of Social Epidemiology, Graduate School of Medicine, Science Frontier Laboratory, Kyoto University, Floor 2, Yoshida-konoe-cho, Sakyo-ku, Kyotoshi, Kyoto Japan; 3 Department of Social Preventive Medical Sciences, Center for Preventive Medical Sciences, Chiba University, 1-33, Yayoicho, Inage-ku, Chiba, Chiba Japan; 4 Department of Gerontological Evaluation, Center for Gerontology and Social Science, National Center for Geriatrics and Gerontology, 7 Chome 430, Moriokacho, Obu, Aichi Japan; 5 Department of Community Health and Preventive Medicine, Hamamatsu University School of Medicine, 1-20-1 Handayama, Higashi-ku, Hamamatsu, Shizuoka Japan; 6 Design Research Institute, Chiba University, 1-19-1, Bunka, Sumida-ku, Tokyo, Japan

**Keywords:** Depressive symptoms, Geographical unit, Japan, Multilevel analysis, Municipality, Neighborhood, Older adults, Rural, Social capital, Urban

## Abstract

**Background:**

Rurality can reflect many aspects of the community, including community characteristics that may be associated with mental health. In this study, we focused on geographical units to address multiple layers of a rural environment. By evaluating rurality at both the municipality and neighborhood (i.e., a smaller unit within a municipality) levels in Japan, we aimed to elucidate the relationship between depression and rurality. To explore the mechanisms linking rurality and depression, we examined how the association between rurality and depression can be explained by community social capital according to geographical units.

**Methods:**

We used cross-sectional data from the 2016 wave of the Japan Gerontological Evaluation Study involving 144,822 respondents aged 65 years or older residing in 937 neighborhoods across 39 municipalities. The population density quintile for municipality-level rurality and the quintile for the time required to reach densely inhabited districts for neighborhood-level rurality were used. We calculated the prevalence ratios of depressive symptoms by gender using a three-level (individual, neighborhood, and municipality) Poisson regression. Community social capital was assessed using three components: civic participation, social cohesion, and reciprocity.

**Results:**

The prevalence of depressive symptoms was higher in municipalities with lower population density than those with the highest population density; the ratios were 1.22 (95% confidence intervals: 1.15, 1.30) for men and 1.22 (1.13, 1.31) for women. In contrast, when evaluating rurality at the neighborhood level, the prevalence of depressive symptoms was 0.9 times lower for men in rural areas; no such association was observed for women. In rural municipalities, community civic participation was associated with an increased risk of depressive symptoms. In rural neighborhoods, community social cohesion and reciprocity were linked to a lower risk of depressive symptoms.

**Conclusions:**

The association between rurality and depression varied according to geographical unit. In rural municipalities, the risk of depression may be higher for both men and women, and the presence of an environment conducive to civic participation may contribute to a higher risk of depression, as observed in this study. The risk of depression in men may be lower in rural neighborhoods in Japan, which may be related to high social cohesion and reciprocity.

**Supplementary Information:**

The online version contains supplementary material available at 10.1186/s12942-021-00296-8.

## Background

Suicide is an important global public health issue that results in approximately 703,000 deaths annually [1]. Depression is recognized as an important risk factor for suicide [2, 3]. It is also a risk factor for physical diseases, such as cardiovascular disease and diabetes, and is the leading global cause of disability [4]. Both suicide and depression have been reported to be associated with rurality or urbanicity, but the directions of the associations are not consistent among studies. Some studies from high-income countries have shown that depression is more prevalent in urban areas than in rural areas, whereas no such association has been observed for low- and middle-income countries [5–8]. In addition, suicide rates have shown to be higher in rural areas than in urban areas in various countries [9–11]. The differences in findings across studies may be due to the differences in the processes of the onset of the two outcomes and the measurements of the health outcomes and of rurality/urbanicity (hereinafter referred to as rurality for simplicity).

A focus on geographical units to evaluate rurality may be beneficial for understanding the mechanisms linking the characteristics of residence and mental health. Rurality is an important area-level factor that encompasses many aspects of a community [12]. The measures of rurality observed at different levels of aggregation can reflect different contextual features, and different putative mechanisms may be implicated [13–15]. For example, given that a municipality is the smallest definable political/administrative unit, disadvantages observed in rural municipalities in terms of public service policies and other specific conditions may be associated with poor mental health-related outcomes. Because the municipal population can be expected to contribute to municipal tax revenue and, in turn, the amount and quality of public service supply and human resources, municipality-level rurality in terms of underpopulation may reflect these varying material environments as a consequence of political decisions made by the municipal government. However, even if the population is large, if they are spread out over a large area of land, that is, if the population density is low, then the efficiency of the use of public resources will be low.

In addition to the overall population density of the municipality, geographical variations in population density within the municipality should also be considered. Highly populated central areas in a municipality, referred to as densely inhabited districts (DIDs), often have a high concentration of community functions necessary for daily life. In contrast, residents of neighborhoods that are far from DIDs within a municipality suffer more from the disadvantages of rural contexts, including limited access to public resources, such as public transportation and opportunities for social activities (e.g., job and group activities). Rural neighborhoods, that is, communities distant from DIDs, can also be expected to have other characteristics. First, rural neighborhoods are likely to base their economic activities more on primary industries (e.g., farming) rather than secondary and tertiary industries. Second, rural neighborhoods may have specific functions in their community social capital, including the senses of mutual trust and reciprocity, related to the types of communal farming activities and the need to help one another in daily life [16]. Hence, neighborhood rurality may be associated with mental health independent of the rurality of the municipality to which the neighborhood belongs, and rurality in a municipality and neighborhood may influence residential mental health in different ways. In one cohort study involving all adults in Sweden, municipality-level rurality was associated with suicide rates in men, and individual socioeconomic status explained the excess risk of suicide in rural municipalities but not at the neighborhood level [17]. However, to the best of our knowledge, no study has investigated the associations between rurality and depression when evaluating rurality by community size (i.e., municipalities and neighborhoods).

The urban/rural differences in the prevalence of depression among Asian countries are inconsistent. Reports from China and Myanmar [18] showed a higher prevalence of depression in rural areas than in urban areas, while reports from India [19], South Korea [20], Taiwan [21], and Vietnam [22] showed the opposite or no statistically significant differences. Investigation of the rural/urban gap on depression prevalence in Japan has been limited to those conducted in a single prefecture [23, 24]. Studies that have evaluated the differences in suicide rates in Japan among rural and urban settings have had an ecological design at the prefectural or municipal level [25–29]. Because of the limitations of such a study design, it was not possible to analyze individual and regional effects separately or discuss them separately at the municipality level and link small regional-level factors to mental health-related outcomes.

**Fig. 1:**
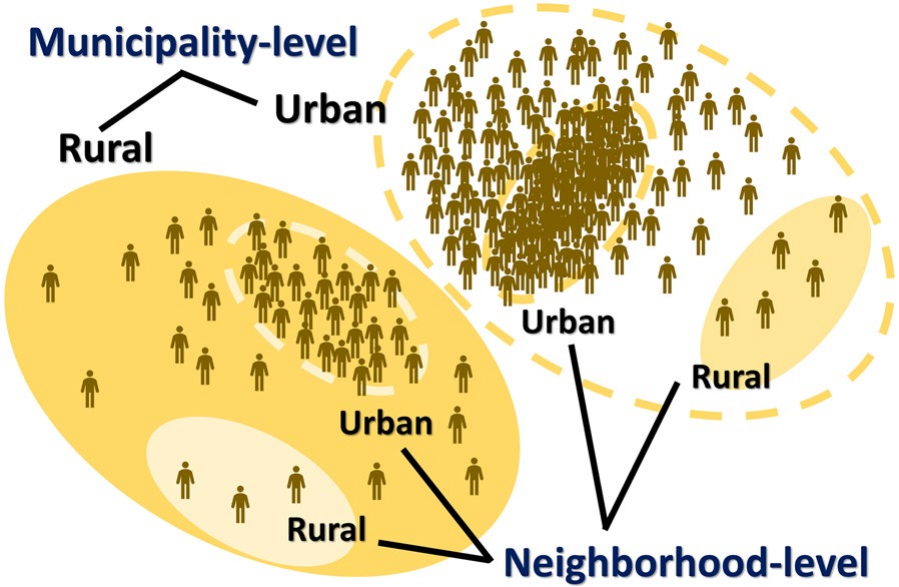
Conceptual figure showing the definitions of rurality/urbanicity by two regional levels in this study. Rurality was assessed at both the municipality and the neighborhood (i.e., a smaller unit within a municipality) levels

With this in mind, our study was designed to evaluate rurality at both the municipality and neighborhood (i.e., a smaller unit within a municipality) levels in Japan (Fig. 1). In Japan, the most aged society in the world, there are many farming neighborhoods located in mountainous areas that are far from the DIDs. In addition, to explore the potential differential mechanisms that link rurality to depression by aggregation unit, we focused on community social capital, a factor that has not been widely investigated in relation to urban–rural differences in depression. To achieve this, we examined how the association between rurality and depression can be explained by community social capital according to geographical units.

## Methods

### Data collection

Cross-sectional data were obtained from the 2016 wave of the Japan Gerontological Evaluation Study (JAGES), a large-scale, population-based collaborative study of voluntarily participating municipalities. The data collection period spanned October 3, 2016, to December 5, 2016, inclusive, with an additional period of data collection in January 2017 for approximately 2000 people residing in a municipality. The JAGES involved the distribution of an anonymous self-administered mail survey in cooperation with different municipalities across Japan. The questionnaire was sent to residents in participating municipalities aged 65 years or older who were not certified as needing public long-term care insurance. The 2016 wave consisted of individuals living in 39 municipalities in 18 prefectures out of the 47 total prefectures in Japan, including the northernmost prefecture (Hokkaido) and the Kyusyu region (Kumamoto prefecture) in the southern area. Municipalities of various population sizes are included, ranging from approximately 1000 to 3.7 million people. The proportion of older adults ranged from 20.5% to 50.4% (the overall proportion of older adults in Japan was 27.3% in 2016 [30]). A map of the study area is available on the JAGES website [31].

In the larger municipalities (n = 22), the participants were randomly selected by a multistage sampling process, while in the smaller municipalities (n = 17), all eligible individuals were included. In total, 279,661 questionnaires were mailed to potential participants, and 196,431 were returned, which equated to a response rate of 70.2%. However, this figure inadvertently included some people certified as needing public long-term care insurance; after excluding these individuals, data for 180,021 individuals were available for analysis. The data have a three-stage hierarchical structure: individuals, school districts (neighborhoods), and municipalities. In Japan's administrative classification, municipalities (*shi, cho/machi,* or *son/mura*) are the smallest local public entities and are positioned a level below prefectures (*to, do, fu,* or *ken*).

To evaluate rurality at the municipality level, we obtained data on municipal population density from the population census. To evaluate rurality at the neighborhood level, we collected data on the time required to reach a DID using the government census of agriculture and forestry for 2015 obtained from the website of the Ministry of Agriculture, Forestry, and Fisheries [32]. We used these data to ensure the proportional distribution of data in each JAGES district.

### Measurements

#### Outcome: depressive symptoms

We used the Japanese short version of the Geriatric Depression Scale (GDS-15) to evaluate depressive symptoms in our cohort. The GDS-15 was developed to assess depressive symptoms in self-administered surveys and consists of 15 items with binary yes (scored 1)/no (scored 0) answer options, resulting in a total score ranging from 0 to 15, with higher scores indicating more depressive symptoms. In this study, we used a score of 6 or more to categorize depressive symptoms, which was used to indicate moderate symptoms in an earlier validation study performed on older Japanese adults [33] and has been reported to be highly associated with suicidal ideation [34].

#### Rurality

**Fig. 2:**
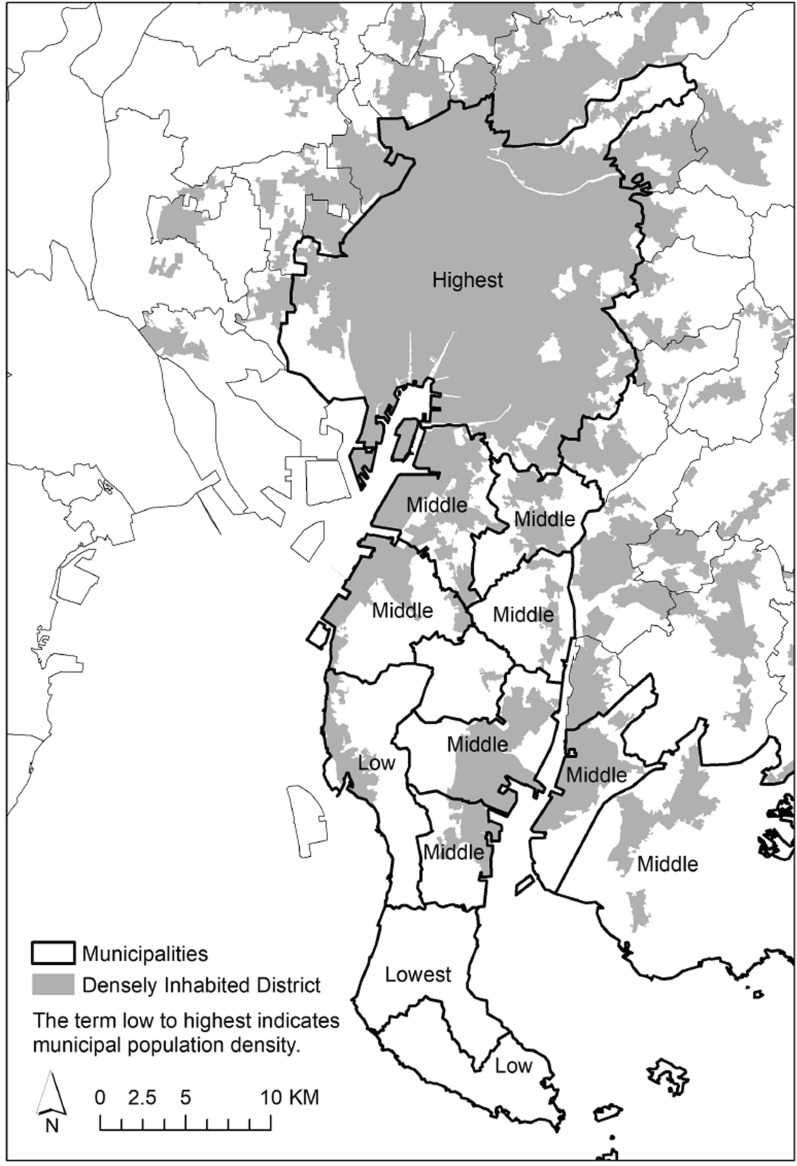
Municipal population density and densely inhabited districts. Municipalities that participated in the study from Chita Peninsula, which is among the study areas, are shown as examples

Because there is no universal definition of rurality, which can include multiple dimensions, such as geographical, political, and cultural characteristics [35], defining rurality according to the study purpose is recommended [36]. In this study, we adopted the following two variables to define rurality: population density quintile (for municipality-level rurality) and quintile of the time required to reach the DID (for neighborhood-level rurality; Fig. 2). The latter variable was defined as the amount of time it takes to travel from the center of a rural community to the center of a DID by the transportation mode (e.g., car, bus, or train) typically used by residents. A study that compared multiple rurality/urbanicity indicators found that population density and accessibility (e.g., number of people that can be reached within a certain travel time) were the most sensitive proxy indicators for identifying the relationship between rurality and suicide [37].

#### Community social capital

We used the health-related community social capital scale [38] to assess three components of community social capital (i.e., levels of community civic participation, social cohesion, and reciprocity) at the neighborhood level. The level of community civic participation was quantified by summing each individual’s participation in any three types of community groups (volunteer groups, sports groups, and hobbies) or activities in which the frequency of participation was once a month or more. Levels of social cohesion in the community were measured by summing the percentage of those who answered “very” or “moderately” to three HR-CSC items: trust (“Do you think that people living in your area can be trusted in general?”), perceptions of others’ intentions to help (“Do you think that people living in your area try to help others in most situations?”), and attachment to the residential area (“How attached are you to the area in which you live?”); other potential answers to these items included “neutral,” “slightly,” and “not at all.” The third component, levels of community reciprocity, was measured by summing the percentage of individuals who received emotional support (“Is there someone who listens to your concerns and complaints?”), provided emotional support (“Do you listen to others’ concerns and complaints?”), or received instrumental support (“Is there someone who looks after you when you are sick and confined to bed for a few days?”). The levels of community civic participation, community social cohesion, and community reciprocity were standardized in our cohort [38].

### Covariates

To improve the robustness of our statistical analysis, we adjusted the data from participants according to their age groups: 65–74 years, 75–84 years, and ≥ 85 years. As individual socioeconomic status may be both a confounder and a mediator in the relationship between rurality and depression, we conducted additional analyses adjusting for individual sociodemographic status as follows: years of education (< 9 years or not, which is equivalent to graduating from junior high school), equalized household income tertiles (high, middle, or low), marital status (having a spouse or not), and living alone or not [39–43].

### Statistical analyses

A total of 35,199 respondents were excluded from our analyses for the following reasons: missing valid values for age (n = 262), gender (n = 30), and residential address (n = 7); living in a very small neighborhood of 50 residents or less (n = 3,255; this was an exclusion criterion to remove neighborhoods with large standard errors in area-level social capital measurements); and a lack of outcome data (n = 31,645). We used a three-level (individual, neighborhood, and municipality) Poisson regression to calculate the prevalence ratios for depressive symptoms. To avoid overestimation of the calculated prevalence ratios, a Poisson regression with robust variance was used to model depressive symptoms, which was regarded as a frequently occurring outcome [44]. We stratified all of the performed analyses by gender because a previous study that examined the relationship between rurality and suicide found different associations for men and women [45]. Statistical analyses were performed using STATA [46] including the *mepoisson* command in STATA/MP 15.1. We incorporated random intercepts at the neighborhood and municipality levels into the analyses.

We could not find a statistical package that could conduct multiple imputations using a three-level model; therefore, we created dummy variables for the missing covariate data and modeled them. First, we analyzed a null (empty) model to evaluate the variance of the random parameters. We then created Model 1, which incorporated municipality-level rurality, neighborhood-level rurality, and age, followed by Models 2–4, which incorporated the variables of Model 1 as well as community social capital (civic participation, social trust, and reciprocity). To evaluate the general contextual effects in our data, we calculated the median rate ratios from the estimated variance of the random parameters, which represented the median relative change in the prevalence of depressive symptoms when comparing two randomly selected residents chosen from two different groups: one with a higher prevalence of depressive symptoms and the other with a lower prevalence of depressive symptoms [47].

To assess the differential effects of neighborhood-level rurality and municipality-level rurality, we tested the interaction term between them (adjusted for age). To perform a sensitivity analysis, rather than using the above rurality measure, we used municipal population density centered on the grand mean as municipality-level rurality and population density centered within the cluster for neighborhood-level rurality.

## Results

In total, data from 69,660 men and 75,162 women living within 937 neighborhoods across 39 municipalities were analyzed. As shown in Table 1, depression was more prevalent in areas with lower population densities among both men and women at the municipal level. In contrast, there was no marked difference in the prevalence of depressive symptoms according to neighborhood-level rurality. Table 2 shows that with respect to community civic participation at the municipality level, lower population density was associated with less citizen participation (mean: 0.86–0.60), while at the neighborhood level, there was slightly lower citizen participation in the school districts that took the longest time to reach a DID. However, this effect was rarely seen at the municipality level (mean: 0.81–0.72). Furthermore, Table 2 also shows that at both the municipality and neighborhood levels, rural areas were associated with higher levels of social cohesion. As shown in Additional file 5: Fig. S1, in rural municipalities, there were more districts that took a longer time to reach DID and had less community civic participation. In addition, compared to rural municipalities, urban municipalities had more social participation overall, regardless of the rurality of the school district.

**Table 1:** Descriptive statistics of the variables included in the study

	Men		Women	
	Participants (n)	Prevalence of depressive symptoms (%)	Participants (n)	Prevalence of depressive symptoms (%)
Age				
65–74	41,265	16.3	44,033	15.2
75–84	24,268	18.3	26,502	17.9
≥ 85	4127	22.6	4627	24.5
Population density (indicator for municipality-level rurality)				
Highest	15,610	16.4	16,180	15.5
High	13,661	18.3	15,043	17.0
Middle	16,188	16.1	17,023	14.8
Low	12,045	18.9	13,179	18.5
Lowest	12,156	17.9	13,737	18.5
Time to reach the DID (indicator for neighborhood-level rurality)				
Shortest	13,740	18.0	15,080	16.8
Short	11,003	17.7	11,931	15.8
Middle	16,832	17.3	18,032	16.6
Long	14,603	16.8	15,291	16.4
Longest	13,482	17.2	14,828	17.8

**Table 2:** Descriptive statistics of community social capital (civic participation, social cohesion, and reciprocity) by rurality

	Number of school districts	Civic participation^a^		Social cohesion^a^		Reciprocity^a^	
		Mean	(SD)	Mean	(SD)	Mean	(SD)
Population density (municipality-level rurality)							
Highest	394	0.86	(0.16)	1.56	(0.15)	1.97	(0.049)
High	259	0.81	(0.15)	1.53	(0.15)	1.96	(0.051)
Middle	87	0.82	(0.13)	1.63	(0.11)	2.00	(0.023)
Low	116	0.72	(0.15)	1.63	(0.12)	1.99	(0.038)
Lowest	81	0.60	(0.16)	1.67	(0.12)	1.99	(0.033)
Time to reach the DID (neighborhood-level rurality)							
Shortest	349	0.81	(0.16)	1.52	(0.15)	1.96	(0.051)
Short	125	0.84	(0.14)	1.59	(0.12)	1.98	(0.038)
Middle	185	0.83	(0.16)	1.58	(0.15)	1.98	(0.051)
Long	140	0.80	(0.18)	1.61	(0.12)	1.99	(0.034)
Longest	138	0.72	(0.21)	1.68	(0.13)	1.99	(0.037)

*SD* standard deviation

^a^Evaluated in school districts. Unstandardized value

The null model of the multilevel Poisson regression analysis confirmed that the prevalence ratio of depressive symptoms varied slightly between municipalities and neighborhoods. The median rate ratios for municipalities and neighborhoods were 1.13 and 1.04 in men and 1.16 and 1.01 in women, respectively (Tables 3, 4). When only neighborhood-level rurality and age were included in the model, the median rate ratio was almost unchanged (data not shown), but when municipality-level rurality was added, the median rate ratio for municipalities decreased to 1.10 in men and 1.11 in women. The variances in depressive symptoms among municipalities and among neighborhoods were partially explained by civic participation (Model 2).

**Table 3:** Prevalence ratios [95% confidence intervals] of depressive symptoms among men: the results of multilevel analysis

	Null	Model 1		Model 2		Model 3		Model 4	
Municipality-level factors									
Population density (ref. highest)									
High		1.10 [0.95,1.28]		1.09 [0.98,1.21]		1.09 [0.96,1.24]		1.08 [0.95,1.22]	
Middle		0.99 [0.89,1.11]		0.94 [0.86,1.04]		1.04 [0.92,1.18]		1.07 [0.96,1.19]	
Low		1.22 [1.15,1.30]		1.08 [0.99,1.18]		1.24 [1.14,1.36]		1.26 [1.18,1.35]	
Lowest		1.16 [1.06,1.26]		0.96 [0.88,1.05]		1.20 [1.10,1.33]		1.16 [1.07,1.27]	
Neighborhood-level factors									
Time to the DID (ref. shortest)									
Short		0.97 [0.88,1.06]		0.98 [0.90,1.06]		0.99 [0.90,1.09]		1.00 [0.93,1.08]	
Middle		0.94 [0.86,1.04]		0.97 [0.91,1.04]		0.97 [0.90,1.04]		0.98 [0.91,1.05]	
Long		0.91 [0.83,0.99]		0.92 [0.87,0.99]		0.94 [0.87,1.02]		0.96 [0.89,1.04]	
Longest		0.90 [0.81,1.00]		0.89 [0.83,0.96]		1.00 [0.90,1.10]		0.96 [0.88,1.06]	
Community social capital									
Civic participation				0.87 [0.85,0.90]					
Social cohesion						0.88 [0.86,0.90]			
Reciprocity								0.90 [0.88,0.91]	
Individual-level factors									
Age (ref. 65–74)									
75–84		1.13 [1.09,1.17]		1.13 [1.09,1.17]		1.13 [1.10,1.17]		1.13 [1.09,1.17]	
≥ 85		1.38 [1.30,1.48]		1.39 [1.31,1.49]		1.40 [1.32,1.49]		1.39 [1.30,1.48]	
Random-effect part of the model									
Between municipality variance^a^	0.016 (0.004)	0.009 (0.003)		0.005 (0.002)		0.007 (0.003)		0.007 (0.002)	
Median rate ratio	1.13	1.10		1.07		1.09		1.08	
Between neighborhood variance^a^	0.002(0.002)	0.001(0.002)		0.000(0.000)		0.000(0.000)		0.000(0.000)	
Median rate ratio	1.04	1.03		1.00		1.00		1.00	

^a^Standard errors in parentheses

**Table 4:** Prevalence ratios [95% confidence intervals] of depressive symptoms among women: the results of multilevel analysis

	Null	Model 1		Model 2		Model 3		Model 4	
Municipality-level factors									
Population density (ref. highest)									
High		1.09 [0.99,1.21]		1.07 [0.99,1.17]		1.08 [0.98,1.20]		1.08 [0.97,1.19]	
Middle		0.96 [0.86,1.06]		0.92 [0.82,1.02]		0.99 [0.89,1.10]		0.99 [0.90,1.09]	
Low		1.22 [1.13,1.31]		1.09 [1.00,1.19]		1.23 [1.12,1.35]		1.24 [1.15,1.32]	
Lowest		1.16 [1.04,1.29]		1.00 [0.89,1.12]		1.20 [1.07,1.34]		1.16 [1.04,1.29]	
Neighborhood-level factors									
Time to the DID (ref. shortest)									
Short		0.95 [0.88,1.03]		0.96 [0.90,1.02]		0.97 [0.90,1.05]		0.97 [0.90,1.04]	
Middle		0.99 [0.91,1.08]		1.02 [0.96,1.09]		1.01 [0.95,1.09]		1.02 [0.95,1.09]	
Long		0.95 [0.89,1.02]		0.97 [0.91,1.03]		0.99 [0.93,1.05]		0.99 [0.93,1.05]	
Longest		0.99 [0.92,1.06]		0.97 [0.90,1.05]		1.07 [0.98,1.16]		1.03 [0.96,1.10]	
Community social capital									
Civic participation				0.89 [0.87,0.91]					
Social cohesion						0.91 [0.90,0.93]			
Reciprocity								0.94 [0.92,0.96]	
Individual-level factors									
Age (ref. 65–74)									
75–84		1.17 [1.13,1.23]		1.17 [1.12,1.22]		1.18 [1.13,1.23]		1.17 [1.12,1.22]	
≥ 85		1.60 [1.50,1.71]		1.60 [1.50,1.70]		1.61 [1.51,1.72]		1.60 [1.50,1.70]	
Random-effect part of the model									
Between municipality variance^a^	0.023 (0.006)	0.012 (0.004)		0.006 (0.002)		0.010 (0.003)		0.010 (0.003)	
Median rate ratio	1.16	1.11		1.07		1.10		1.10	
Between neighborhood variance^a^	0.000(0.002)	0.000(0.000)		0.000(0.000)		0.000(0.000)		0.000(0.000)	
Median rate ratio	1.01	1.00		1.00		1.00		1.00	

^a^ Standard errors in parentheses

Among men, the prevalence of depressive symptoms was approximately 1.2 times higher in municipalities with lower population density than in municipalities with the highest population density (Model 1, Table 3). The prevalence of depressive symptoms in men was 0.90 times lower in neighborhoods with a longer time required to reach the DID than in neighborhoods with the shortest time required (Model 1, Table 3). Low levels of civic participation in rural municipalities explained their excess risk of depression (Model 2). High levels of social cohesion and reciprocity in rural neighborhoods were linked to a lower risk of depressive symptoms (Models 3 and 4).

In women, the prevalence of depressive symptoms was approximately 1.2 times higher in municipalities with lower population density than in municipalities with the highest population density (Model 1, Table 4). The prevalence of depressive symptoms in women did not vary according to neighborhood rurality (Model 1, Table 4), but civic participation explained the excess risk of depression in rural municipalities (Model 2).

Individual sociodemographic status also partly explained the high risk of depressive symptoms in rural municipalities but did not explain the differences in neighborhoods (Additional file 1: Table S1). Our sensitivity analyses showed that the results of the regression models using alternative rurality measures were mostly similar to those of our original models (Additional files 2, 3: Table S2, S3). No consistent pattern was observed for the interaction between municipality-level rurality and neighborhood-level rurality (Additional file 6: Fig. S2).

## Discussion

Our data indicate that the prevalence of depressive symptoms is about 1.2 times higher for both genders in rural municipalities. The excess risk of depressive symptoms in rural municipalities is partly explained by neighborhood-level civic participation but not by other social capital domains; this accounted for 3–4% of the variance in depressive symptoms among municipalities and 1–3% of the variance among neighborhoods. In contrast, at the neighborhood level, compared to urban neighborhoods, the prevalence of depressive symptoms was 0.9 times lower for men in rural neighborhoods, whereas no such association was observed for women. The lower risk is partly explained by neighborhood-level social cohesion and neighborhood-level reciprocity but not by neighborhood-level civic participation.

The differences in the association between rurality and depression and the mechanisms underlying the variation across geographical units have received little attention among studies of the urban–rural depression gap. A study performed in Ghana and South Africa found no substantial urban–rural differences in the strength of the association between social capital and depression [48]. In a report from China that focused on the impact of rapid urbanization on social capital and health, the association between social capital and depression varied by period and rurality [49]. Psychological variables, including social capital, social capital satisfaction, and self-esteem, also play a role in depression among the elderly, as shown by Korea Welfare Panel Study data [50] as well as data from the China Family Panel Studies [51].

Community civic participation assessed at the neighborhood level partly explained the high level of depression in rural municipalities. The presence of a social and material environment that makes it difficult for residents to participate in social activities, such as fewer participation opportunities and limited infrastructure, may contribute to a high risk of depression in rural municipalities. Similar results have been obtained by studies conducted among older adults in China, and in particular, an association between high depression risk and low social participation has been reported [52]. Limited availability of public transportation in rural municipalities could reduce social participation [53], resulting in poor mental health [54–56]. Moreover, rural municipalities tend to have less civic participation overall, regardless of the variability in the characteristics of the neighborhood within the municipalities. In fact, municipalities with a low population density include many neighborhoods with long travel times to reach DIDs (Additional file 5: Fig. S1). On the other hand, even in neighborhoods from which reaching DIDs takes a long time, the level of civic participation tended to be higher if the neighborhoods were located in municipalities with high population density.

Based on the potential difficulty in social environments related to municipality-level rurality, in rural municipalities, the frequency and variability of social gathering opportunities may be limited, making it difficult for people to find gatherings that interest them. In Japan, driven by the strong recommendation and financial incentive schemes of the central government, municipal governments have provided support for local people to set up “community salons” (voluntary social gathering activities) for older people. However, compared to urban municipalities, rural municipalities have fewer salons [57]. Regional differences in local norms for participating in hobbies and other social activities may also explain the findings of this study. For example, while conducting this research, several government officials in rural municipalities introduced the following statements by residents: “I can’t go to the salon because (if I go there) neighbors may say I am playing without doing any work.” Compared to urban municipalities, in rural municipalities, more people participated in “semi-formal” local associations such as senior citizens’ associations and community councils, but fewer people participated in informal activities, as mentioned above [58].

On the other hand, to interpret the results showing a lower prevalence of depressive symptoms in rural neighborhoods than in urban neighborhoods and indicating that neighborhood-level cohesion and reciprocity are partly explained the rurality/depression association, it may be instructive to focus on the characteristics of community social capital stemming from the sociodemographic characteristics of rural neighborhoods. In JAGES, rural neighborhoods had a high number of people with extensive farming experience and fewer people living alone (Additional file 4: Table S4). Neighborhood cohesion and reciprocity fostered by communal work through farming may have a preventive effect against depression among farmers. In the farming community, there are many types of communal work, such as the management of waterways and prevention of damage from wildlife. For example, in areas with low farm density, where the availability of job-related mutual support is limited, the prevalence of depression among farmers is high [59]. Nonetheless, the effects of fostered social capital through farming could spill over to non-farmers in the same neighborhood [60]. In contrast, reverse causation should also be considered. That is, depressed people may be less connected to the community and live alone, which is another known risk factor of depression, and they may reside in more convenient city centers. However, even after adjusting for individual socioeconomic status, the trend toward less depression in rural neighborhoods remained (Additional file 1: Table S1).

The policy implications of this study are twofold. First, the evaluation of the prevalence of depression by area, in combination with regional characteristics, including rurality, is essential. If depression is more prevalent in rural areas than in urban areas, developing interventions that match the characteristics of the area is required, taking into account the possible meanings of the rurality assessed at each level, as discussed, the rurality evaluated at large units can represent policy-relevant social structures, and that evaluated at small units tends to reflect cultural and normative aspects of social relations. For example, the JAGES initiative, which provided the data used in this study, has provided participating municipalities with a tool for visualizing differences in health and related social factors among and within municipalities [61]. In promoting knowledge translation and evidence-based community-based care [62, 63], the researcher’s assistance provided to municipal offices has a positive impact on residents’ health and socioeconomic equity [64]. The findings of this study, which was conducted in Japan, the most aged society in the world, could be applied to other countries, including Asian nations, where many countries are ageing at a faster rate than Japan. For example, nationwide population aging is likely to induce population decline and economic inactivity in rural areas, leading to socioeconomic disadvantages, limited access to healthcare and other public services [65], and poor mental health [9, 10, 12] for residents. Consequently, population aging may cause urban/rural disparities in mental health. A positive aspect is that in small communities, such as the neighborhood level in this study, the potential preventive effect of community social cohesion and reciprocity on depression may exist [66]. The effective use of social capital in small communities may buffer the structural disadvantages in rural areas. These may be more applicable to countries with similar cultural/industrial features, including those where rice farming is common [67].

This study has several strengths and limitations that should be highlighted. The main strength of this study is that it used data from residents and not hospital patients; thus, the results are not biased by the differences between urban and rural areas with respect to the ease of diagnosis due to healthcare access. In addition, our approach of large-scale sampling in the general population residing in various areas is unlikely to be biased by geographical variations in access to healthcare and allowed us to consider multiple regional units simultaneously. Regarding limitations, first, the generalizability of our findings may be limited by the coverage of the sampling frame, given that our dataset was not representative of the entire Japanese population. Second, as the data collected in this study were self-reported, this may have given rise to various forms of bias, such as recall bias and social desirability bias. Third, this manuscript focuses on one country (Japan), and the results may not be generalizable to other countries, particularly those with different social infrastructure, cultures, and mental healthcare provision. Rurality is a diverse and relative concept, and each country’s definition of rurality and the contexts reflected by those definitions can vary greatly [6]. Fourth, we did not analyze air pollution levels, proximity to roads, green areas, or traffic noise because of a lack of access to these data. Fifth, we were not able to identify and discuss the diversity of rural communities.

## Conclusions

The results of this study suggest that the association between rurality and depression varies according to the geographical unit analyzed. In rural municipalities, the risk of depression may be higher for both men and women, and the presence of an environment conducive to civic participation may contribute to a higher risk of depression, as observed in this study. The risk of depression in men may be lower in rural neighborhoods in Japan, which may be related to high social cohesion and reciprocity.

## Supplementary Information

**Additional file 1: Table S1.** Prevalence ratios [95% confidence intervals] of depressive symptoms: results of the multilevel analysis using individual sociodemographic characteristics

**Additional file 2: Table S2.** Prevalence ratios [95% confidence intervals] of depressive symptoms among men: results of the sensitivity analyses

**Additional file 3: Table S3.** Prevalence ratios [95% confidence intervals] of depressive symptoms among women: results of the sensitivity analyses

**Additional file 4: Table S4.** Proportion of men and women living alone and of farmers by neighborhood-level rurality

**Additional file 5: Figure S1.** Distribution of mean community civic participation evaluated by neighborhood through municipality-level rurality and neighborhood-level rurality.

**Additional file 6: Figure S2.** Estimated prevalence of depressive symptoms with 95% confidence intervals by gender: cross-level interaction between municipality-level rurality and neighborhood-level rurality on depressive symptoms. The estimates were derived from a three-level multilevel Poisson regression adjusted for age as well as municipality-level rurality and neighborhood-level rurality.

## Data Availability

The datasets used and/or analyzed during the current study are available from the corresponding author on reasonable request.

## Abbreviations

DID: Densely inhabited district
JAGES: Japan Gerontological Evaluation Study
