# Supplementary material for: Differences in depressive symptoms by rurality in Japan: a cross-sectional multilevel study using different aggregation units of municipalities and neighborhoods (JAGES)
Source: Int J Health Geogr. 2021 Sep 26;20:42. doi: 10.1186/s12942-021-00296-8 (PMC8474726; doi:10.1186/s12942-021-00296-8)
Supplement: Supplementary file 1 — Additional file 1: Table S1. Prevalence ratios [95% confidence intervals] of depressive symptoms: results of the multilevel analysis using individual sociodemographic characteristics [file 12942_2021_296_MOESM1_ESM.docx]

| Supplemental Table 1. Prevalence ratios [95% confidence intervals] of depressive symptoms: the results of the multilevel analysis using individual sociodemographic characteristics | | | | | | | | |
| --- | --- | --- | --- | --- | --- | --- | --- | --- |
|  | Men | | | | Women | | | |
|  | Model 1 | | Model 1' | | Model 1 | | Model 1' | |
| *Municipality-level factors* |  |  |  |  |  |  |  |  |
| **Population density (ref. highest)** |  |  |  |  |  |  |  |  |
| High | 1.10 | [0.95,1.28] | 1.08 | [0.98,1.19] | 1.09 | [0.99,1.21] | 1.07 | [0.97,1.17] |
| Middle | 0.99 | [0.89,1.11] | 0.95 | [0.86,1.05] | 0.96 | [0.86,1.06] | 0.91 | [0.83,0.99] |
| Low | 1.22 | [1.15,1.30] | 1.12 | [1.04,1.20] | 1.22 | [1.13,1.31] | 1.12 | [1.05,1.20] |
| Lowest | 1.16 | [1.06,1.26] | 1.01 | [0.92,1.10] | 1.16 | [1.04,1.29] | 1.03 | [0.92,1.15] |
| *Neighborhood-level factors* |  |  |  |  |  |  |  |  |
| **Time to the DID (ref. shortest)** | |  |  |  |  |  |  |  |
| Short | 0.97 | [0.88,1.06] | 0.99 | [0.93,1.05] | 0.95 | [0.88,1.03] | 0.95 | [0.89,1.02] |
| Middle | 0.94 | [0.86,1.04] | 0.96 | [0.90,1.02] | 0.99 | [0.91,1.08] | 0.99 | [0.93,1.06] |
| Long | 0.91 | [0.83,0.99] | 0.93 | [0.88,0.99] | 0.95 | [0.89,1.02] | 0.95 | [0.89,1.02] |
| Longest | 0.90 | [0.81,1.00] | 0.90 | [0.83,0.97] | 0.99 | [0.92,1.06] | 0.97 | [0.90,1.03] |
| *Individual-level factors* |  |  |  |  |  |  |  |  |
| **Education: > 9 years (ref. <=9 years)** |  |  | 1.28 | [1.24,1.32] |  |  | 1.24 | [1.20,1.28] |
| **Income (Ref. T3 [High])** |  |  |  |  |  |  |  |  |
| T2 (Middle) |  |  | 1.65 | [1.58,1.72] |  |  | 1.48 | [1.42,1.54] |
| T1 (Low) |  |  | 2.34 | [2.22,2.47] |  |  | 2.09 | [1.99,2.21] |
| **Living alone: yes (ref. no)** |  |  | 1.48 | [1.40,1.56] |  |  | 1.13 | [1.06,1.20] |
| **Having a spouse: no (ref: yes)** |  |  | 1.44 | [1.37,1.52] |  |  | 1.15 | [1.11,1.19] |
| **Age (ref. 65–74)** |  |  |  |  |  |  |  |  |
| 75–84 | 1.13 | [1.09,1.17] | 1.05 | [1.01,1.08] | 1.17 | [1.13,1.23] | 1.02 | [0.98,1.07] |
| >= 85 | 1.38 | [1.30,1.48] | 1.20 | [1.13,1.27] | 1.60 | [1.50,1.71] | 1.31 | [1.22,1.40] |
| *Random-effect part of the model* |  |  |  |  |  |  |  |  |
| Between municipality variance* | 0.009(0.003) | | 0.003(0.002) | | 0.012(0.004) | | 0.008(0.003) | |
| *Median rate ratio* | 1.10 |  | 1.05 |  | 1.11 |  | 1.09 |  |
| Between neighborhood variance* | 0.001(0.002) | | 0.000(0.000) | | 0.000(0.000) | | 0.000(0.000) | |
| *Median rate ratio* | 1.03 |  | 1.00 |  | 1.00 |  | 1.00 |  |
| *Standard errors in parentheses |  |  |  |  |  |  |  |  |

DID: Densely Inhabited District
