# Supplementary material for: Differences in depressive symptoms by rurality in Japan: a cross-sectional multilevel study using different aggregation units of municipalities and neighborhoods (JAGES)
Source: Int J Health Geogr. 2021 Sep 26;20:42. doi: 10.1186/s12942-021-00296-8 (PMC8474726; doi:10.1186/s12942-021-00296-8)
Supplement: Supplementary file 2 — Additional file 2: Table S2. Prevalence ratios [95% confidence intervals] of depressive symptoms among men: results of the sensitivity analyses [file 12942_2021_296_MOESM2_ESM.docx]

| Supplemental Table 2. Prevalence ratios [95% confidence intervals] of depressive symptoms among men: the results of the sensitivity analyses | | | | | | | | |  |
| --- | --- | --- | --- | --- | --- | --- | --- | --- | --- |
|  |  |  |  |  |  |  |  |  |  |
|  | Null | Model 1 |  | Model 2 |  | Model 3 |  | Model 4 |  |
| *Municipality-level factors* |  |  |  |  |  |  |  |  |  |
| **Population density centered using grand mean (1000/km^2^)** | | 0.99 | [0.98,1.01] | 1.02 | [1.00,1.03] | 0.98 | [0.97,0.99] | 0.98 | [0.97,1.00] |
| *Neighborhood-level factors* |  |  |  |  |  |  |  |  |  |
| **Population density centered within cluster (1000/km^2^)** | | 1.01 | [1.01,1.02] | 1.01 | [1.01,1.02] | 1.00 | [1.00,1.01] | 1.00 | [1.00,1.01] |
| **Community social capital** |  |  |  |  |  |  |  |  |  |
| Civic participation |  |  |  | 0.88 | [0.85,0.90] |  |  |  |  |
| Social cohesion |  |  |  |  |  | 0.88 | [0.86,0.90] | |  |
| Reciprocity |  |  |  |  |  |  |  | 0.89 | [0.88,0.91] |
| *Individual-level factors* |  |  |  |  |  |  |  |  |  |
| **Age (ref. 65–74)** |  |  |  |  |  |  |  |  |  |
| 75–84 |  | 1.13 | [1.09,1.17] | 1.13 | [1.09,1.17] | 1.13 | [1.10,1.17] | 1.13 | [1.09,1.17] |
| >= 85 |  | 1.39 | [1.30,1.48] | 1.39 | [1.30,1.48] | 1.4 | [1.32,1.49] | 1.39 | [1.30,1.48] |
| *Random-effect part of the model* | |  |  |  |  |  |  |  |  |
| Between municipality variance* | 0.016(0.004) | 0.016(0.004) | | 0.009(0.002) |  | 0.012(0.004) | | 0.010(0.003) | |
| *Median rate ratio* | 1.13 | 1.13 |  | 1.09 |  | 1.11 |  | 1.10 |  |
| Between neighborhood variance* | 0.002(0.002) | 0.001(0.002) | | 0.000(0.000) |  | 0.000(0.000) | | 0.000(0.000) | |
| *Median rate ratio* | 1.04 | 1.04 |  | 1.00 |  | 1.00 |  | 1.00 |  |
|  |  |  |  |  |  |  |  |  |  |
| *Standard errors in parentheses |  |  |  |  |  |  |  |  |  |
