# Supplementary material for: Differences in depressive symptoms by rurality in Japan: a cross-sectional multilevel study using different aggregation units of municipalities and neighborhoods (JAGES)
Source: Int J Health Geogr. 2021 Sep 26;20:42. doi: 10.1186/s12942-021-00296-8 (PMC8474726; doi:10.1186/s12942-021-00296-8)
Supplement: Supplementary file 3 — Additional file 3: Table S3. Prevalence ratios [95% confidence intervals] of depressive symptoms among women: results of the sensitivity analyses [file 12942_2021_296_MOESM3_ESM.docx]

| Supplemental Table 3. Prevalence ratios [95% confidence intervals] of depressive symptoms among women: the results of the sensitivity analyses | | | | | | | | |  |
| --- | --- | --- | --- | --- | --- | --- | --- | --- | --- |
|  |  |  |  |  |  |  |  |  |  |
|  | Null | Model 1 |  | Model 2 |  | Model 3 |  | Model 4 |  |
| *Municipality-level factors* |  |  |  |  |  |  |  |  |  |
| **Population density centered using grand mean (1000/km^2^)** | | 0.99 | [0.97,1.00] | 1.01 | [0.99,1.02] | 0.98 | [0.96,0.99] | 0.98 | [0.97,0.99] |
| *Neighborhood-level factors* |  |  |  |  |  |  |  |  |  |
| **Population density centered within cluster (1000/km^2^)** | | 1.00 | [0.99,1.01] | 1.00 | [1.00,1.01] | 1.00 | [0.99,1.00] | 1.00 | [0.99,1.00] |
| **Community social capital** |  |  |  |  |  |  |  |  |  |
| Civic participation |  |  |  | 0.89 | [0.87,0.92] |  |  |  |  |
| Social cohesion |  |  |  |  |  | 0.91 | [0.90,0.93] | |  |
| Reciprocity |  |  |  |  |  |  |  | 0.94 | [0.92,0.95] |
| *Individual-level factors* |  |  |  |  |  |  |  |  |  |
| **Age (ref. 65–74)** |  |  |  |  |  |  |  |  |  |
| 75–84 |  | 1.18 | [1.13,1.23] | 1.17 | [1.12,1.22] | 1.18 | [1.13,1.23] | 1.17 | [1.12,1.22] |
| >= 85 |  | 1.60 | [1.50,1.71] | 1.60 | [1.50,1.70] | 1.61 | [1.51,1.72] | 1.6 | [1.50,1.71] |
| *Random-effect part of the model* | |  |  |  |  |  |  |  |  |
| Between municipality variance* | 0.023(0.006) | 0.019(0.005) | | 0.009(0.002) |  | 0.017(0.004) | | 0.015(0.004) | |
| *Median rate ratio* | 1.16 | 1.14 |  | 1.10 |  | 1.13 |  | 1.12 |  |
| Between neighborhood variance* | 0.000(0.002) | 0.000(0.000) | | 0.000(0.000) |  | 0.000(0.000) | | 0.000(0.000) | |
| *Median rate ratio* | 1.01 | 1.00 |  | 1.00 |  | 1.00 |  | 1.00 |  |
|  |  |  |  |  |  |  |  |  |  |
| *Standard errors in parentheses |  |  |  |  |  |  |  |  |  |
