# Supplementary material for: Differences in depressive symptoms by rurality in Japan: a cross-sectional multilevel study using different aggregation units of municipalities and neighborhoods (JAGES)
Source: Int J Health Geogr. 2021 Sep 26;20:42. doi: 10.1186/s12942-021-00296-8 (PMC8474726; doi:10.1186/s12942-021-00296-8)
Supplement: Supplementary file 4 — Additional file 4: Table S4. Proportion of men and women living alone and of farmers by neighborhood-level rurality [file 12942_2021_296_MOESM4_ESM.docx]

Supplemental Table 4. Proportion of men and women living alone and of farmers by neighborhood-level rurality

|  | Living alone | |  | Longest occupation* | | |
| --- | --- | --- | --- | --- | --- | --- |
|  | Number of respondents | Proportion of people living alone (%) |  | Number of respondents | Proportion of farmers (%) | |
| **Men** | | | | | |  |
| Time to reach the DID (neighborhood-level rurality) | | | | | |  |
| Shortest | 13275 | 14.1 |  | 12676 | 0.7 | |
| Short | 10531 | 10.0 |  | 10108 | 2.3 | |
| Middle | 16127 | 9.7 |  | 15440 | 2.7 | |
| Long | 14013 | 8.7 |  | 13417 | 4.6 | |
| Longest | 12839 | 9.3 |  | 12105 | 11.3 | |
|  | | | | | |  |
| **Women** | | | | | |  |
| Time to reach the DID (neighborhood-level rurality) | | | | | |  |
| Shortest | 14368 | 25.7 |  | 13502 | 0.5 | |
| Short | 11296 | 19.7 |  | 10531 | 2.4 | |
| Middle | 17047 | 19.5 |  | 15914 | 2.8 | |
| Long | 14406 | 16.7 |  | 13364 | 5.0 | |
| Longest | 13813 | 17.3 |  | 12753 | 8.9 | |

*The occupation that respondents engaged in longest across their lifetime. Respondents who answered ‘agriculture, forestry, or fisheries’ were categorized as “farmers.” All others were categorized as “non-farmers” (professional/technical; managerial; clerical; sales/services; skilled labor; self-employment other than agriculture, forestry, and fisheries; other).

DID: Densely Inhabited District
