# Supplementary material for: Differences in depressive symptoms by rurality in Japan: a cross-sectional multilevel study using different aggregation units of municipalities and neighborhoods (JAGES)
Source: Int J Health Geogr. 2021 Sep 26;20:42. doi: 10.1186/s12942-021-00296-8 (PMC8474726; doi:10.1186/s12942-021-00296-8)
Supplement: Supplementary file 5 — Additional file 5: Figure S1. Distribution of mean community civic participation evaluated by neighborhood through municipality-level rurality and neighborhood-level rurality. [file 12942_2021_296_MOESM5_ESM.docx]

Supplemental Figure 1. Distribution of mean community civic participation evaluated by neighborhood through municipality-level rurality and neighborhood-level rurality.

**
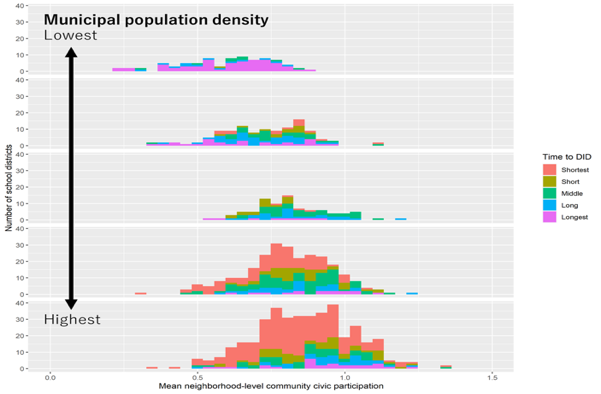
**
