# Supplementary material for: Differences in depressive symptoms by rurality in Japan: a cross-sectional multilevel study using different aggregation units of municipalities and neighborhoods (JAGES)
Source: Int J Health Geogr. 2021 Sep 26;20:42. doi: 10.1186/s12942-021-00296-8 (PMC8474726; doi:10.1186/s12942-021-00296-8)
Supplement: Supplementary file 6 — Additional file 6: Figure S2. Estimated prevalence of depressive symptoms with 95% confidence intervals by gender: cross-level interaction between municipality-level rurality and neighborhood-level rurality on depressive symptoms. The estimates were derived from a three-level multilevel Poisson regression adjusted for age as well as municipality-level rurality and neighborhood-level rurality. [file 12942_2021_296_MOESM6_ESM.docx]

| Supplemental Figure 2. Estimated prevalence of depressive symptoms with 95% confidence intervals by gender: cross-level interaction between municipality-level rurality and neighborhood-level rurality on depressive symptoms. The estimates were derived from a three-level multilevel Poisson regression adjusted for age as well as municipality-level rurality and neighborhood-level rurality.  Time to DID  (Neighborhood-level rurality) |  |
| --- | --- |
|  |  |
